# Clinical and histological patterns and treatment of pyoderma gangrenosum

**DOI:** 10.11604/pamj.2020.36.59.12329

**Published:** 2020-06-02

**Authors:** Radia Chakiri, Hanane Baybay, Asmae El Hatimi, Salim Gallouj, Taoufiq Harmouch, Fatima Zohra Mernissi

**Affiliations:** 1 Department of Dermatology, University Hospital Hassan II, Fez, Morocco,; 2 Department of Anatomopathology, University Hospital Hassan II, Fez, Morocco

**Keywords:** Pyoderma gangrenosum, neutrophilic dermatosis, systemic disease

## Abstract

Pyoderma gangrenosum (PG) is a rare inflammatory neutrophilic dermatosis for which accurate epidemiological data are limited and therapy remains a challenge. The primary study’s aim was to examine all cases of PG observed in our department over a 6-year period in order to describe the relevant characteristics and outcome under therapy. Fourteen patients were included (5 women, 9 men). The average age of our patients was 40,15 years. The classical, ulcerative form was found in 10 cases (71.42%), the pustular form in 4 cases (27.57%) and PG was multifocal in 4 cases. The PG was located preferentially to the lower limbs. Histological examination was realized in all patients and objectified inflammatory infiltrate composed of polymorphonuclear neutrophils in all cases with vasculitis in 4 cases. Six patients (42.85%) had associated disease at diagnosis of PG, including inflammatory bowel disease in two cases (14.28%), a blood disease in 2 cases (14.28%), lymph node tuberculosis and inflammatory arthritis in 1 case (7%). The most frequent first-line treatments were oral corticosteroids (7 cases) and other treatments used were colchicine in 2 cases, topical corticosteroids in 3 cases with good clinical evolution. Our study confirms that PG is a rare disease, associated in almost half of cases with systemic disease already present at diagnosis; in our Moroccan background, it is most often inflammatory bowel disease, hematological or solid cancer and tuberculosis.

## Introduction

Pyoderma gangrenosum (PG), first described by Brocq in 1916 and named by Brunsting *et al*. in 1930, is a rare non infectious neutrophilic dermatosis that typically presents as destructive cutaneous ulcerations that usually appear on the legs [1, 2]. PG can occur at any age; however, it generally presents in the second to fifth decades of life. A century after its first description, we have found out that it was not related to an infection despite its original definition, but we still do not fully understand the pathophysiology of PG. Currently, the loss of innate immune regulation and altered neutrophil chemotaxis are believed to be involved to some extent and considering its association with other auto-inflammatory diseases such as Crohn disease and Behcet´s disease, PG is now included within the spectrum of systemic auto inflammatory diseases [1,3]. The hallmark of the disease is painful ulcerations that can affect any area of the body but are most commonly found in the lower legs.

In the classical type, skin lesions arise suddenly as painful, tender, erythematous papules, plaques, nodules or pustules that rapidly progress to expanding ulcers with characteristic violaceous undermined edges and a necrotic base. Healing of these ulcers usually results in characteristic atrophic cribriform scars [1, 4, 5]. A diagnostic criterion has been also defined [6]; however, it has not been uniformly accepted according to a study [7]. Thus, diagnosis is solely based on clinical findings and exclusion of other ulcerating skin diseases [1, 5]. PG occurs in association with an underlying disease such as inflammatory bowel disease (IBD), inflammatory arthritis, hematological disorders and solid malignancies in 50% to 70% of the cases [4].

Thus, investigations are also necessary to determine whether there is a treatable systemic, associated disorder. Remaining cases are rather idiopathic or related to other factors such as trauma or surgery [5,7]. Recent studies of PG associated genetic syndromes may provide insight into the pathogenesis of PG and may help develop specific therapies against new targets [3,5]. PG is a relatively rare disease that makes it difficult to obtain results from randomized controlled studies and treatments are traditionally individualized according to patient compliance and associating systemic diseases. Thus, there is no standard treatment protocol for PG patients [1, 4, 7]. The primary aim of our study was to examine all cases of PG observed in our department over a 6-year period in order to describe the relevant characteristics and outcome under therapy.

## Methods

We present a monocentric prospective, observational study included PG patients who were treated between 2009 and 2015 in the Department of Dermatology, University Hospital Hassan II, Fez, Morocco. Cases were identified by screening for patients with a discharge diagnosis of PG that was based on clinical findings and histopathological features consistent with PG together with investigations to exclude other causes including infections, tumors or vasculitis. Age, gender, clinical and histological findings, demographics, comorbidities, therapeutic modalities and outcome were recorded.

## Results

**Figure 1:**
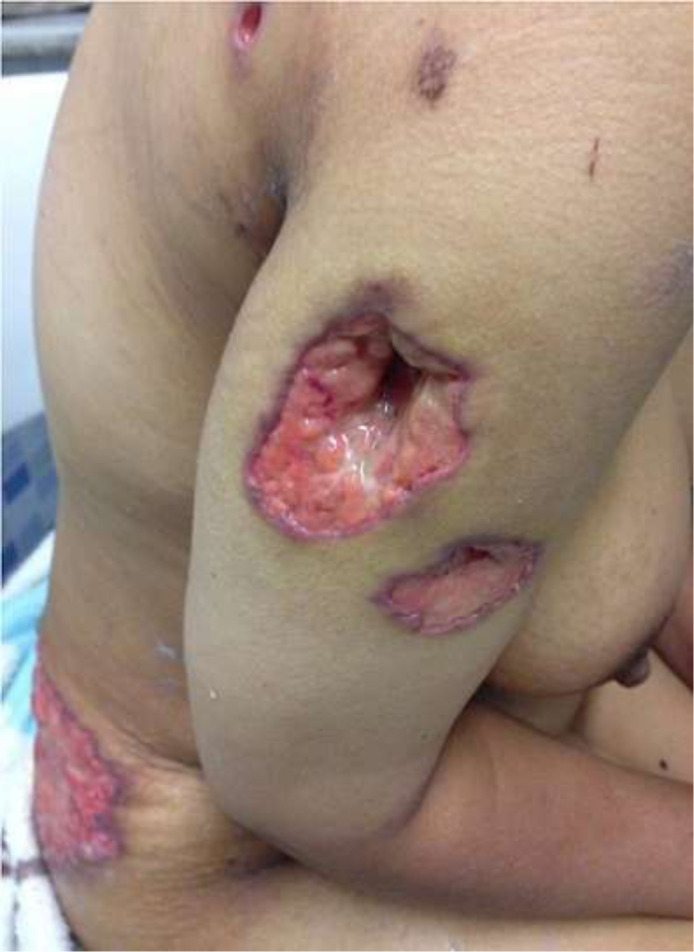
disseminated ulcerative pyoderma gangrenosum

**Figure 2:**
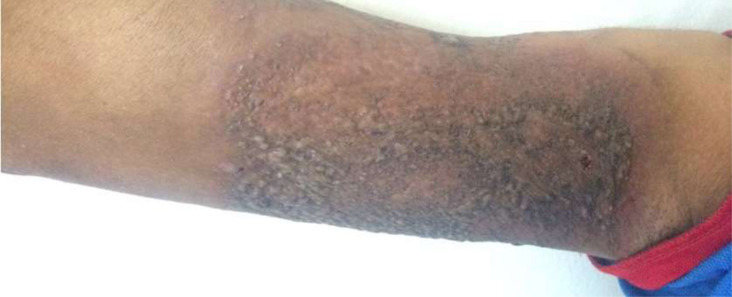
pustular pyoderma gangrenosum

**Figure 3:**
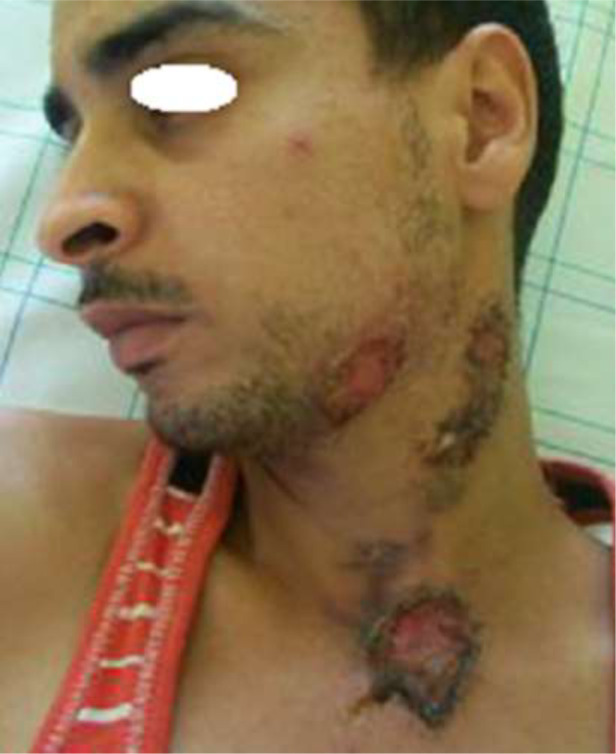
ulcerative pyoderma gangrenosum of the neck

**Clinical and histopathological characteristics:** the clinical characteristics of the patients at diagnosis of PG are summarized in Table 1. For the 14 patients included (5 women, 9 men; sex ratio H / F = 0.4), the average age at diagnosis was 40.15 years (range: 11-70 years). The classical, ulcerative form was found in 10 cases (71.42%) (Figure 1) and the pustular form in 4 cases (27.57%) (Figure 2). The PG was located preferentially to the lower limbs in 10 cases, trunk, upper limb and neck (Figure 3) in one case each. Histological examination showed a dense infiltrate of neutrophil in all patients, this infiltrate was associated with vasculitis in four cases and lymphoplasmacytic infiltrate in five cases.

**Table 1:** clinical and histopathological characteristics of patients

Case Nº	Age/gender	Associated diseases	Type of PG	Localization	Histology
1	55/M	-	Ulcerative	Legs	Infiltrate of neutrophil
2	29/M	-	Ulcerative	Legs	Neutrophilic and lymphoplasmacytic infiltrate
3	23/F	-	Pustular	Legs	Infiltrate of neutrophil, vasculitis
4	48/M	Multiple myeloma	Pustular and ulcerative	Legs	Neutrophilic and lymphoplasmacytic infiltrate, vasculitis
5	64/M	Bowel disease	Ulcerative	Legs	Neutrophilic and lymphoplasmacytic infiltrate
6	40/M	-	Ulcerative	Legs	Infiltrate of neutrophil
7	40/F	-	Ulcerative	Trunk, neck	Neutrophilic and lymphoplasmacytic infiltrate, vasculitis
8	70/M	-	Ulcerative	Legs	Infiltrate of neutrophil
9	36/F	Rheumatoid arthritis chronic lymphocytic leukemia	Ulcerative	Legs, thighs and buttocks	Neutrophilic and lymphoplasmacytic infiltrate, vasculitis
10	11/M	-	Pustular	Legs	Infiltrate of neutrophil
11	38/M	-	Pustular	Upper limbs, legs	Neutrophilic and lymphoplasmacytic infiltrate
12	34/F	Pregnancy	Ulcerative	Legs	Infiltrate of neutrophil
13	34/F	-	Ulcerative	Legs	Neutrophilic and lymphoplasmacytic infiltrate
14	29/M	Bowel disease	Ulcerative	Upper limbs	Infiltrate of neutrophil

**Associated diseases:** six patients (42,85%) had associated disease at diagnosis of PG, including inflammatory bowel disease in 2 cases (14.28%), a blood disease in 2 cases (14.28%), lymph node tuberculosis and inflammatory arthritis in one case each (7%). In our series one patient was pregnant at 6 month.

**Treatments and follow-up:** the patients included in the study were followed for varying periods ranging from 3 months to 4 years. Treatment with oral steroids (Prednisone: 0.5-1mg / kg / day) was prescribed first line in 9 cases (64.28%), alone or combined with topical treatment. Topical corticosteroids were used in first line in three cases (21.42%). The remaining two cases were treated with colchicine 1 mg/day.

## Discussion

Pyoderma gangrenosum may occur at any age but typically affects those between 20 and 50 years (51.8%), as described by our study and in previous studies [8-10]. In our series we noted a male predominance; however, in most series reported in the literature pyoderma gangrenosum affects women more than men. The clinical characteristics of PG in our study are comparable to published data, with a predominance of the classical ulcerative form. Other clinical variant, bullous, pustular, vegetative and peristomal types are possible but rare. Lesions can appear on any part of the body, but ulcers of the classical subtype favor the lower extremities in up to 85.7% of the patients [11-13], as was the case for 71.42% of our patients. The reason for this specific location is not known [13]. Binus *et al*. [14] found that almost one third of the patients had comorbid conditions such as diabetes and/or peripheral vascular disease and raised the question that these 2 diseases might play a role in and contribute to the development of PG and worsen local healing processes, especially on the lower leg.

The diagnosis of PG relies on clinical signs first and is supported by biopsy for histopathology. Knowledge of the patient's history for possible underlying disease and specific investigations based on that background are necessary. Therefore, diagnosis is made by exclusion of other possible disorders. No laboratory parameter for PG is available. The histopathology of PG is no specific and changes with the stage of lesion. The initial lesions show a deep suppurative folliculitis with dense neutrophilic infiltrate. In about 40% of cases, leukocytoclastic vasculitis is present as was in four of our cases. PG with (necrotizing) granulomatous inflammation has been described [15-17]. An associated disease, similar to those observed in other neutrophilic dermatosis, was found in 42.85% of our patients, which is consistent with the literature, where the frequency of pathological associations is between 33 and 84% [1, 18-20].

IBD was identified in 14.28% of cases (20% to 30% in the literature), a blood disease in 14,28% of cases (15% to 25% in the literature), inflammatory arthritis in 7% of cases (20% to 30% in the literature) and ganglionic tuberculosis in 7% of our cases (extremely rare association in the literature). However, thyroid diseases (including cancer of the thyroid) were very rare [18]. Because the etiology of the disease is not being understood, there is no specific and uniformly effective therapy for PG. The aim of the treatment is to reduce pain and to promote wound healing by reducing inflammation with anti-inflammatory and immunosuppressive agents, thereby improving the quality of life of PG patients.

Thus for extended and active lesions, treatments which have shown efficacy in several case series are oral corticosteroid therapy with prednisone doses ranging from 40 to 120 mg per day in adults and cyclosporine (average dose of 5 to 7 mg / kg / day) [21-23]. Our study had some limitations related to the assessment of a small number of PG patients and patient recruitment through the only dermatology department which was originally a selection bias. Thus, the results of this study need to be confirmed by multicenter studies with large number of patients and especially by involving other specialists for patient recruitment.

## Conclusion

Our study confirms that the PG is a rare disease, associated in almost half of cases with systemic disease already present at diagnosis; it is most often IBD, blood disorders, solid cancers and tuberculosis in our Moroccan background.

### What is known about this topic

- PG a rare inflammatory neutrophilic dermatosis;
- Association with systemic diseases.

### What this study adds

- The first study of PG in Morocco;
- Tuberculosis association with PG in our background.
